# Supplementary material for: Acceptance of a Novel, Highly Palatable, Calorically Dense, and Nutritionally Complete Diet in Dogs with Benign and Malignant Tumors
Source: Vet Sci. 2023 Feb 11;10(2):148. doi: 10.3390/vetsci10020148 (PMC9961485; doi:10.3390/vetsci10020148)
Supplement: Supplementary file 1 [file vetsci-10-00148-s001.zip › vetsci-2031436-supplementary.pdf]

Supplemental Material

Table of contents **Page**

**Table S1.** Adverse events	2
**Surveys**	3
**Pet Owner Food Evaluation at Enrollment**	3
**Pet Owner Food Evaluation: Days 1 through 10, 14, 21, 28**	6
**Pet Quality of Life Questionnaire**	8
**Pet Owner Exit Questionnaire**	10
**Vet Exit Study Food Evaluation**	12
**Stool Quality Questionnaire**	14
**Body Fat Index Scale**	16

Table S1. Adverse events

Adverse event description	Total, n	Study food related, n	Not Study food related, n	Life-threatening, n	Severe, n	Moderate, n	Mild, n
Hyporexia	1	0	1	0	1	0	0
Hematochezia	1	0	1	1	0	0	0
Vomiting/nausea	11	0	11	0	2	0	9
Soft stool/diarrhea/flatulence	11	0	11	0	0	2	9
Hypertriglyceridemia/ hypercholesterolemia/serum lipemia	13	0	13	0	1	2	10
Increased defecation	1	0	1	0	0	0	1
Weight gain	4	0	4	0	0	0	4
Increased panting/drooling	4	0	4	0	0	1	3
Decreased appetite	8	2	6	0	1	0	7
Pancreatitis	1	0	1	0	0	0	1
Increased thirst	3	0	3	0	0	1	2
**Total**	**58**	**2**	**56**	**1**	**5**	**6**	**46**

Surveys

Pet Owner Food Evaluation at Enrollment

Please reflect on your pet's eating habits over the last 4 weeks to answer the following questions.

1. Do you make each meal the same way? (For example "no" if you feed wet in the morning and dry at night or "yes" if you mix wet and dry at each meal or "yes" if you only feed dry or only feed wet.)

   Yes or No

   If No, *we need to query about each meal using the number of meals they said they feed in q2*

   During Meal 1 each day what do you offer your dog?

   - ❑ Dry food only
   - ❑ Wet food only
   - ❑ A combination of wet and dry in the same bowl
   - ❑ Wet and dry in separate bowls

   Meal 2

   - ❑ Dry food only
   - ❑ Wet food only
   - ❑ A combination of wet and dry in the same bowl
   - ❑ Wet and dry in separate bowls

   (no of options would reflect answer to question 2)

   If Yes, *we only need to ask once*
   At each meal what do you offer your dog?

   - ❑ Dry food only
   - ❑ Wet food only
   - ❑ A combination of wet and dry in the same bowl
   - ❑ Wet and dry in separate bowls

2. We'd like to know how much you currently offer your dog total in one day. Please turn on the scale, change it to grams and weigh your dog's empty bowl and enter the weight here.

enter weight in grams

3. Zero (tare) the scale. Add the total amount of your current dry food that you typically offer over the course of a day. How much does the bowl weigh now in grams?

Enter the same weight if you did not add any dry food.
Need an invisible data field that is weight of (food+bowl) - (weight of bowl) = weight of dry food.

4. If you feed wet food we now need to weigh that.
   Empty the bowl and now use the bowl that you feed wet food in (could be the same or a different bowl).
   Weigh the bowl that you feed the wet food in. *enter weight in grams*

5. Zero (tare) the scale. Add the total amount of your current wet food that you typically offer over the course of a day. How much does the bowl weigh now in grams?

Enter the same weight if you did not add any wet food.
Need an invisible data field that is weight of (food+bowl) - (weight of bowl) = weight of wet food.

6. Most recently what percent of your current food that you offer does your dog typically eat?
   0-100% slider (0= none and 100% is All)

Please answer the following questions by marking the box which best represents your opinion of each question.

7. **Thinking about the last 2-3 days, how would you rank your dog’s enjoyment of or enthusiasm for eating their food.**

   ❑ Check this box if you did not observe

   1-7 point Emoji Scale inserted here

8. **How would you describe your dog's strength today.**

   ❑ Check this box if you did not observe

Very Weak Very
Strong

9. **How would you describe your dog's energy level today.**

   ❑ Check this box if you did not observe

Very Low Very High

10. **How would you describe your dog’s vitality today**

    ❑ Check this box if you did not observe

Poor Excellent

11. **How would you describe your dog’s hair coat today**

    ❑ Check this box if you did not observe

Dull and Dry Shiny and Soft

12. Are you going to feed the study food starting today?

Yes
No

if yes go to next form
if no go to next form next day

13. How many meals (not including treats) per day do you want to offer your dog?

(drop down 1, 2, 3, 4, 5, 6, 7, 8, 9, 10, more than 10)

Recorder's Signature — Recorder's Signature Date (DD/MM/YYYY)

Investigator's Signature — Investigator's Signature Date (DD/MM/YYYY)

Pet Owner Food Evaluation: Days 1 through 10, 14, 21, 28

Date: (/   / 20   )

Name of Person Interviewed:______________

Today is Day XX. Once you answer how many meals you want to offer your dog today we will inform you how much of the study food to offer your dog in each meal. For days 1 through 7, after the study food meal you can offer your dog a reduced portion of their old food each day as you increase the amount of study food (from days 1 through 7 only because you are gradually transitioning your dog to eat the study food). After day 7, your dog will have completely transitioned to the study food and will be eating only study food.

1. How many meals (not including treats) do you want to offer your dog today?

(drop down 1-10, >10)

Provide the owner with guidance from the Feeding Schedule "Today you are planning on offering [answer from q1] meals . This means you will feed XX g of dry food at each meal. For days 1-7 as you transition to the study food, after your dog completes their study meal you can feed a reduced portion of their previous food" . After day 7, you will only be feeding the study food.

FOR ONE MEAL

3. Please turn on the scale, change it to grams and weigh your dog's empty bowl and enter the weight here.

enter weight in grams

4. Zero (tare) the scale. Add the amount of the study food you are going to feed, if any, in ONE meal. How much does the bowl weigh now in grams?

Enter total weight in grams.

Prelude to automatically calculate weight of dry food added Need an invisible data field that is weight of (food+bowl) - (weight of bowl) = weight of dry food.

5. Did your dog eat everything in the bowl?

IF Yes: next question

IF NO: record weight of the left over food including the bowl = _________ gms.

Prelude to automatically calculate weight of food left by taking away weight of the bowl

Please answer the following questions by marking the box which best represents your opinion of each question.

6. Thinking about the last meal you fed your dog, how would you rank your dog's enjoyment of or enthusiasm for eating their food.

❑ Check this box if you did not observe

1-7 point Emoji Scale inserted here

7. How would you describe your dog's strength today? If the owner is feeding the same day as enrollment, carry over the same answer to this question for Day 1 only.

❑ Check this box if you did not observe

Very Weak Very
Strong

8. How would you describe your dog's energy level today? If the owner is feeding the same day as enrollment, carry over the same answer to this question for Day 1 only.

❑ Check this box if you did not observe

Very Low Very High

9. How would you describe your dog’s vitality today? If the owner is feeding the same day as enrollment, carry over the same answer to this question for Day 1 only.

❑ Check this box if you did not observe

Poor Excellent

If you desire please briefly describe your dog’s eating behavior over the last day:

____

____

____

____
Recorder's Signature Recorder's Signature Date (DD/MM/YYYY)

____
Investigator's Signature Investigator's Signature Date (DD/MM/YYYY)

Pet Quality of Life Questionnaire

Date: (/   /20   )

Name of Person Interviewed:_______________

A) Check the box below at the point that describes how would you agree or disagree with the following statements.

1. My dog vomited in the last 48 hours

❑ Check this box if you do not know

No, not at all   Yes,
frequently

2. My dog is playful today.

❑ Check this box if you did not observe

Strongly disagree   Strongly agree

3. My dog is capable of doing his/her favorite activities today.

❑ Check this box if you did not observe

Strongly disagree   Strongly agree

4. My dog acted like his/her normal self today.

❑ Check this box if you did not observe

Strongly disagree   Strongly agree

5. My dog is joyful today.

❑ Check this box if you did not observe

Strongly disagree Strongly agree

6. My dog enjoyed being near me today.

❑ Check this box if you did not observe

Strongly disagree Strongly agree

7. My dog showed their normal amount of affection for the family today.

❑ Check this box if you did not observe

Strongly disagree Strongly agree

8. My dog enjoyed being petted or touched by the family today.

❑ Check this box if you did not observe

Strongly disagree Strongly agree

9. My dog sleeps well

❑ Check this box if you did not observe

Strongly disagree Strongly agree

Recorder's Signature Recorder's Signature Date (DD/MM/YYYY)

Investigator's Signature Investigator's Signature Date (DD/MM/YYYY)

Pet Owner Exit Questionnaire

Date: (/   /20   )

Name of Person Interviewed:

Please reflect on your pet's eating habits after starting to feed Study Food to answer the following questions.

1. **How would you describe your dog's hair coat today compared to the beginning of study?**

   ❑ Check this box if you did not observe

More Dull and Dry   More Shiny and Soft

2. **Do you feel that your dog eating this food helped your dog retain their muscle?**

   ❑ Check this box if you did not observe

No, not at all   Yes, definitely

3. **Do you feel that your dog had more good or great days while consuming the study food?**

   ❑ Check this box if you did not observe

No, not at all   Yes, definitely

4. **Do you feel that feeding this food during the study enhanced the relationship that you have with your dog?**

   ❑ Check this box if you did not observe

No, not at all   Yes, definitely

5. **Do you feel that offering this food helped extend your dog's life?**

   ❑ Check this box if you did not observe

No, not at all   Yes, definitely

6. Did you feed treats to your dog during this study?
    - ❑ Yes
    - ❑ No
    - ❑ If yes, what did you offer your dog? FREE TEXT FIELD

7. Did you feed supplements to your dog during this study?
    - ❑ Yes
    - ❑ No
    - ❑ If yes, what did you offer your dog? FREE TEXT FIELD

8. Did you feed wet food to your dog during this study?
    - ❑ Yes
    - ❑ No
    - ❑ If yes, what did you offer your dog? FREE TEXT FIELD

9. How has your dog's appetite changed since starting the study?

Significantly worse Significantly better

10. How likely are you to recommend the study food to a friend or relative who has a dog living with cancer?

No, not at all Yes, I definitely would

11. Please briefly describe how your dog's eating behavior during meal time has changed during the study:

12. How would your dog describe the food?

Recorder's Signature Recorder's Signature Date (DD/MM/YYYY)

Investigator's Signature Investigator's Signature Date (DD/MM/YYYY)

Vet Exit Study Food Evaluation

•

Please answer the following questions by marking the box which best represents your opinion of each question.

1. **Did feeding the study food contribute to this pet's quality of life?**

   ❑ Check this box if you did not observe

No, not at all   Yes, definitely

2. Check the box below at the point that best describes **Would you recommend this food to a colleague?**

   ❑ Check this box if you did not observe

No, not at all   Yes, definitely

3. Check the box below at the point that best describes **Do you feel that continued eating extends the life of pets living with cancer?**

   ❑ Check this box if you did not observe

No, not at all   Yes, definitely

4. Check the box below at the point that best describes **How has the skin and coat quality changed since the start of the study**

   ❑ Check this box if you did not observe

Significantly worse   Significantly Improved

5. What did you like best about the food experience with this pet

___

___

Recorder's Signature Recorder's Signature Date (DD/MM/YYYY)

Investigator's Signature Investigator's Signature Date (DD/MM/YYYY)

Stool Quality Questionnaire

WHAT IS THE TYPICAL APPEARANCE OF YOUR DOG'S STOOL *(please see below for Gastrointestinal Grading Sheet for reference).*

For multiple defecations per day; Please report the most common grade observed.

If your dog has multiple stools per day please report the most frequently observed grade.

- ☐ Grade 1: Greater than two-thirds of the feces in the defecation is liquid. The feces has lost all form, appearing as a puddle or squirt.
- ☐ Grade 2: Soft-liquid feces are an intermediate between soft and liquid feces. Approximately equal amounts of feces in a defecation are soft and liquid. Form is similar to thick mud; logs are not visible.
- ☐ Grade 3: Greater than two-thirds of the feces in the defecation are soft. The feces retains enough form to pile and some log shapes are discernable, but have lost their firm cylindrical appearance.
- ☐ Grade 4: Firm-soft feces are an intermediate between the grades of firm and soft and both may appear in a single defecation; that is approximately equal amounts of feces in the defecation is firm or soft.
- ☐ Grade 5: Greater than two-thirds of the feces in the defecation is firm. The feces has a cylindrical shape with little flattening.

Gastrointestinal Grading Sheet

Grade 1

Greater than two-thirds of the feces in a defecation are liquid. The feces have lost all form, appearing as a puddle or squirt.

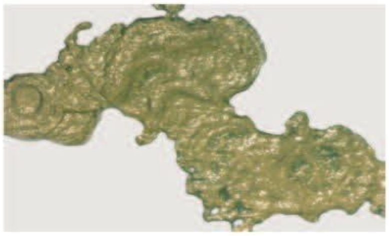

Grade 2

Soft-liquid feces are an intermediate between soft and liquid feces. Approximately equal amounts of feces in a defecation are soft and liquid.

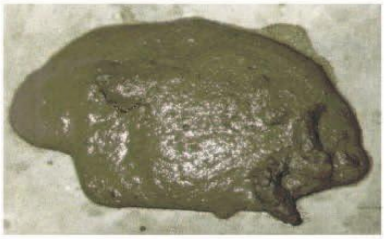

Grade 3

Greater than two-thirds of the feces in a defecation are soft. The feces retain enough form to pile but have lost their firm cylindrical appearance.

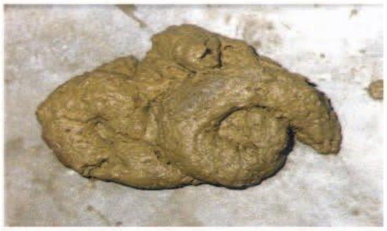

Grade 4

Firm-soft feces are an intermediate between the grades of firm and soft. Approximately equal amounts of feces in a defecation are firm and soft.

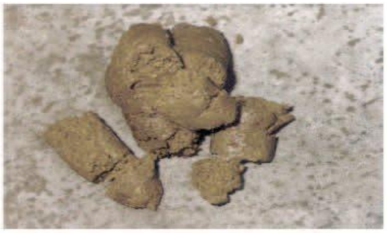

Grade 5

Greater than two-thirds of the feces in a defecation are firm. They have a cylindrical shape with little flattening.

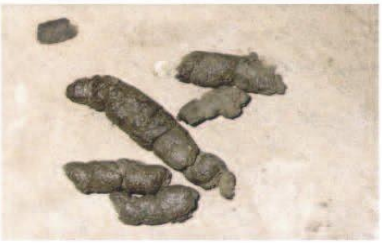

Body Fat Index Scale

Available at: https://www.spca.org.hk/images/PDF/HillsBFIChartCanine.pdf

Hill's™ BFI (Body Fat Index) Risk Chart

20 16-25% Body Fat	30 26-35% Body Fat	40 36-45% Body Fat	50 46-55% Body Fat	60 56-65% Body Fat	70 >65% Body Fat
Low Risk	Mild Risk	Moderate Risk	Serious Risk	Severe Risk	Extreme Risk
**Ribs** Slightly prominent. Easily felt. Thin fat cover.	**Ribs** Slightly to not prominent. Can be felt. Moderate fat cover.	**Ribs** Not prominent. Very difficult to feel. Thick fat cover.	**Ribs** Not prominent. Extremely difficult to feel. Very thick fat cover.	**Ribs** Not prominent. Impossible to feel. Extremely thick fat cover.	**Ribs** Unidentifiable. Impossible to feel. Extremely thick fat cover.
**Shape From Above** Well proportioned lumbar waist.	**Shape From Above** Detectable lumbar waist.	**Shape From Above** Loss of lumbar waist, broadened back.	**Shape From Above** Markedly broadened back.	**Shape From Above** Extremely broadened back.	**Shape From Above** Extremely broadened back, bulging mid-section.
**Shape From the Side** Abdominal tuck present.	**Shape From the Side** Slight abdominal tuck.	**Shape From the Side** Flat to bulging abdomen.	**Shape From the Side** Marked abdominal bulge.	**Shape From the Side** Severe abdominal bulge.	**Shape From the Side** Very severe abdominal bulge.
**Shape From Behind** Clear muscle definition, smooth contour.	**Shape From Behind** Losing muscle definition, rounded appearance.	**Shape From Behind** Rounded to square appearance.	**Shape From Behind** Square appearance.	**Shape From Behind** Square appearance.	**Shape From Behind** Irregular or upside down pear shape.
**Tail Base Bones** Slightly prominent. Easily felt.	**Tail Base Bones** Slightly to not prominent. Can be felt.	**Tail Base Bones** Not prominent. Very difficult to feel.	**Tail Base Bones** Not prominent. Extremely difficult to feel.	**Tail Base Bones** Not prominent. Impossible to feel.	**Tail Base Bones** Unidentifiable.
**Tail Base Fat** Thin fat cover.	**Tail Base Fat** Moderate fat cover.	**Tail Base Fat** Thick fat cover. May have a small fat dimple.	**Tail Base Fat** Very thick fat cover. Fat dimple or fold present.	**Tail Base Fat** Extremely thick fat cover. Large fat dimple or fat fold.	**Tail Base Fat** Extremely thick fat cover. Large fat folds or pads.

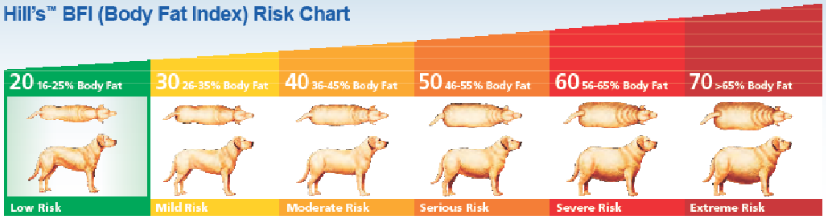

RECOMMENDED BY VETERINARIANS WORLDWIDE™

Developed and validated in partnership with veterinarians at the University of Tennessee.

THE UNIVERSITY of TENNESSEE

3 steps to determine ideal weight

1. Weigh the pet
2. Determine the pet's body fat percentage using images and descriptors on the reverse side
3. Establish ideal weight using this chart

Current Weight (Kg)	IDEAL BODY WEIGHT (Kg) Body Fat % 20	Body Fat % 30	Body Fat % 40	Body Fat % 50	Body Fat % 60	Body Fat % 70
1	1.0	0.9	0.8	0.6	0.5	0.4
2	2.0	1.8	1.5	1.3	1.0	0.8
3	3.0	2.6	2.3	1.9	1.5	1.1
4	4.0	3.5	3.0	2.5	2.0	1.5
5	5.0	4.4	3.8	3.1	2.5	1.9
6	6.0	5.3	4.5	3.8	3.0	2.3
7	7.0	6.1	5.3	4.4	3.5	2.6
8	8.0	7.0	6.0	5.0	4.0	3.0
9	9.0	7.9	6.8	5.6	4.5	3.4
10	10.0	8.8	7.5	6.3	5.0	3.8
12	12.0	10.5	9.0	7.5	6.0	4.5
14	14.0	12.3	10.5	8.8	7.0	5.3
16	16.0	14.0	12.0	10.0	8.0	6.0
18	18.0	15.8	13.5	11.3	9.0	6.8
20	20.0	17.5	15.0	12.5	10.0	7.5
22	22.0	19.3	16.5	13.8	11.0	8.3
24	24.0	21.0	18.0	15.0	12.0	9.0
26	26.0	22.8	19.5	16.3	13.0	9.8
28	28.0	24.5	21.0	17.5	14.0	10.5
30	30.0	26.3	22.5	18.8	15.0	11.3
33	33.0	28.9	24.8	20.6	16.5	12.4
36	36.0	31.5	27.0	22.5	18.0	13.5
39	39.0	34.1	29.3	24.4	19.5	14.6
42	42.0	36.8	31.5	26.3	21.0	15.8
45	45.0	39.4	33.8	28.1	22.5	16.9
48	48.0	42.0	36.0	30.0	24.0	18.0
51	51.0	44.6	38.3	31.9	25.5	19.1
55	55.0	48.1	41.3	34.4	27.5	20.6
59	59.0	51.6	44.3	36.9	29.5	22.1
63	63.0	55.1	47.3	39.4	31.5	23.6
67	67.0	58.6	50.3	41.9	33.5	25.1
71	71.0	62.1	53.3	44.4	35.5	26.6
75	75.0	65.6	56.3	46.9	37.5	28.1
79	79.0	69.1	59.3	49.4	39.5	29.6

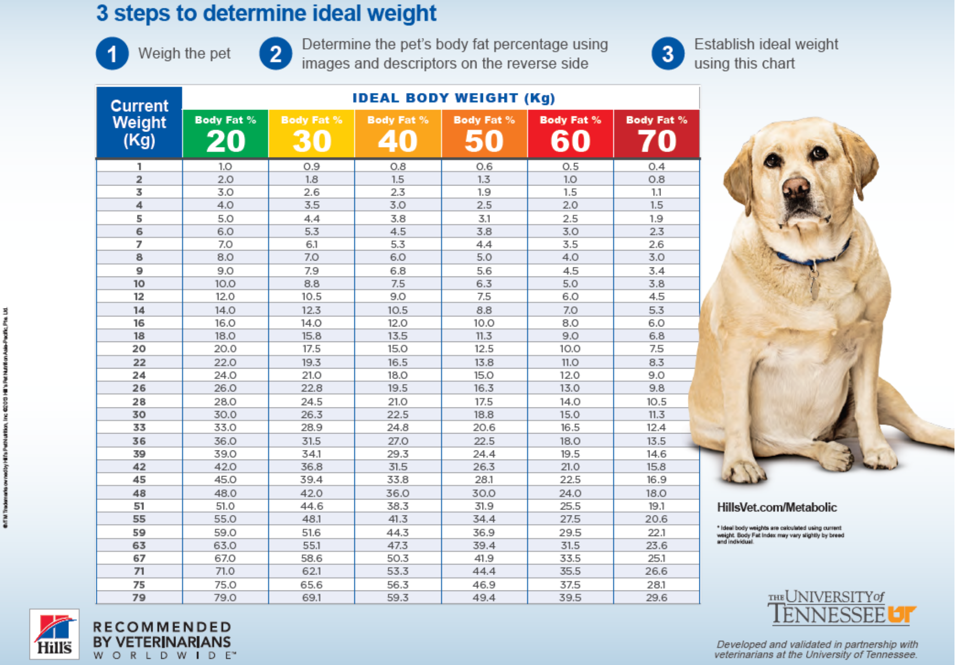

HillsVet.com/Metabolic

* Ideal body weights are calculated using current weight. Body Fat Index may vary slightly by breed and individual.

THE UNIVERSITY of TENNESSEE

Developed and validated in partnership with veterinarians at the University of Tennessee.

RECOMMENDED BY VETERINARIANS WORLDWIDE™
